# *N*-(3-Chloro-4-methyl­phen­yl)succinamic acid

**DOI:** 10.1107/S1600536812007623

**Published:** 2012-02-24

**Authors:** U. Chaithanya, Sabine Foro, B. Thimme Gowda

**Affiliations:** a Department of Chemistry, Mangalore University, Mangalagangotri 574 199, Mangalore, India; b Institute of Materials Science, Darmstadt University of Technology, Petersenstrasse 23, D-64287 Darmstadt, Germany

## Abstract

In the crystal structure of the title compound, C_11_H_12_ClNO_3_, the asymmetric unit contains two independent mol­ecules. The N—H bond in the amide segment is *anti* to the *meta*-Cl atom in the benzene ring, in both molecules. The amide and carboxyl C=O bonds are also *anti* to each other and *anti* to the H atoms on the adjacent –CH_2_ groups. Furthermore, the C=O and O—H bonds of the acid group are in *syn* positions with respect to each other. The dihedral angles between the benzene ring and the amide group are 40.6 (2) and 44.9 (3)° in the two independent molecules. In the crystal, mol­ecules are packed into sheets parallel to the (11-3) plane through O—H⋯O and N—H⋯O hydrogen bonds.

## Related literature

For our studies on the effects of substituents on the structures and other aspects of *N*-(ar­yl)-amides, see: Gowda *et al.* (2000 ▶); Chaithanya *et al.* (2012 ▶). For *N*-(ar­yl)-methane­sulfonamides, see: Gowda *et al.* (2007 ▶). For *N*-chloro­aryl­amides, see: Gowda *et al.* (2003 ▶); Jyothi & Gowda (2004 ▶). For *N*-bromo­aryl­sulfonamides, see: Usha & Gowda (2006 ▶).
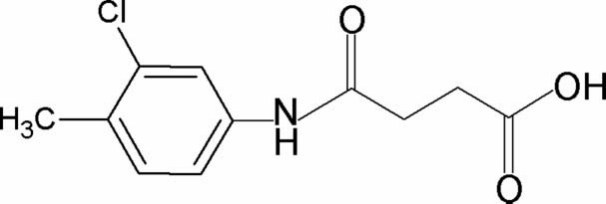


## Experimental

### 

#### Crystal data

C_11_H_12_ClNO_3_
*M* *_r_* = 241.67
Triclinic, 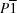

*a* = 6.8788 (9) Å
*b* = 7.9713 (9) Å
*c* = 21.119 (3) Å
α = 86.76 (1)°
β = 86.48 (1)°
γ = 79.45 (1)°
*V* = 1135.1 (3) Å^3^
*Z* = 4
Mo *K*α radiation
μ = 0.33 mm^−1^
*T* = 293 K
0.42 × 0.14 × 0.08 mm

#### Data collection

Oxford Diffraction Xcalibur diffractometer with a Sapphire CCD detector
Absorption correction: multi-scan (*CrysAlis RED*; Oxford Diffraction, 2009 ▶) *T*_min_ = 0.875, *T*_max_ = 0.974
6890 measured reflections
4066 independent reflections
2649 reflections with *I* > 2σ(*I*)
*R*_int_ = 0.028

#### Refinement

*R*[*F* ^2^ > 2σ(*F* ^2^)] = 0.083
*wR*(*F* ^2^) = 0.159
*S* = 1.26
4066 reflections
303 parameters
4 restraints
H atoms treated by a mixture of independent and constrained refinement
Δρ_max_ = 0.23 e Å^−3^
Δρ_min_ = −0.29 e Å^−3^

### 

Data collection: *CrysAlis CCD* (Oxford Diffraction, 2009 ▶); cell refinement: *CrysAlis RED* (Oxford Diffraction, 2009 ▶); data reduction: *CrysAlis RED*; program(s) used to solve structure: *SHELXS97* (Sheldrick, 2008 ▶); program(s) used to refine structure: *SHELXL97* (Sheldrick, 2008 ▶); molecular graphics: *PLATON* (Spek, 2009 ▶); software used to prepare material for publication: *SHELXL97*.

## Supplementary Material

Crystal structure: contains datablock(s) I, global. DOI: 10.1107/S1600536812007623/bt5820sup1.cif

Structure factors: contains datablock(s) I. DOI: 10.1107/S1600536812007623/bt5820Isup2.hkl

Supplementary material file. DOI: 10.1107/S1600536812007623/bt5820Isup3.cml

Additional supplementary materials:  crystallographic information; 3D view; checkCIF report

## Figures and Tables

**Table 1:** Hydrogen-bond geometry (Å, °)

*D*—H⋯*A*	*D*—H	H⋯*A*	*D*⋯*A*	*D*—H⋯*A*
N1—H1N⋯O4^i^	0.85 (2)	2.05 (2)	2.882 (5)	165 (5)
O3—H3O⋯O2^ii^	0.83 (2)	1.83 (2)	2.654 (5)	173 (7)
N2—H2N⋯O1^iii^	0.84 (2)	2.07 (2)	2.891 (5)	165 (5)
O6—H6O⋯O5^iv^	0.83 (2)	1.90 (3)	2.703 (5)	164 (7)

Symmetry codes: (i) 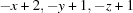; (ii) 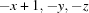; (iii) 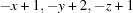; (iv) 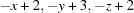.

**Fig. 1.**
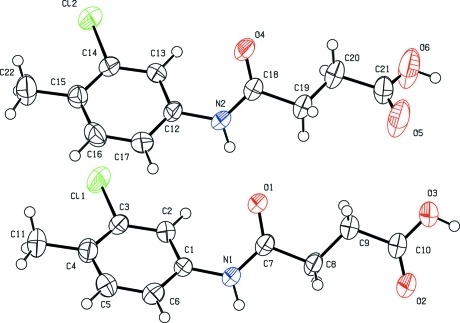
Molecular structure of the title compound, showing the atom labelling scheme and with displacement ellipsoids drawn at the 50% probability level.

**Fig. 2.**
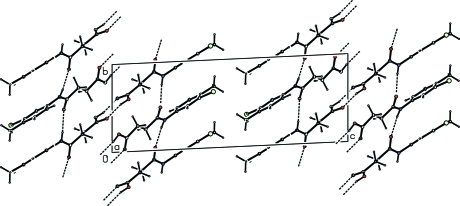
Molecular packing of the title compound with hydrogen bonding shown as dashed lines.

## supplementary crystallographic information

### Comment

As part of our studies on the substituent effects on the structures and other aspects of *N*-(aryl)-amides (Gowda *et al.*, 2000; Chaithanya *et al.*, 2012), *N*-(aryl)-methanesulfonamides (Gowda *et al.*, 2007), *N*-chloroarylsulfonamides (Gowda *et al.*, 2003; Jyothi & Gowda, 2004) and *N*-bromoarylsulfonamides (Usha & Gowda, 2006), in the present work, the crystal structure of *N*-(3-Chloro-4-methylphenyl)succinamic acid has been determined (Fig. 1). The asymmetric unit of the structure contains two independent molecules. The conformations of the N—H bonds in the amide segments are *anti* to the *meta*–chloro atoms in the benzene rings of both the molecules, in contrast to the *syn* conformation observed between the N—H bond and *ortho*–chloro atom in *N*-(2-Chloro-4-methylphenyl)-succinamic acid (I) (Chaithanya *et al.*, 2012).

Further, the conformations of the amide oxygen and the carboxyl oxygen of the acid segments are *anti* to each other and both are *anti* to the H atoms on the adjacent –CH_2_ groups.

The C═O and O—H bonds of the acid groups are in *syn* position to each other, similar to that observed in (I).

The dihedral angles between the phenyl ring and the amide group in the two independent molecules are 40.58 (22)° and 44.93 (27)°, compared to the value of 48.39 (12)° in (I).

In the structure, the pairs of O—H···O and N—H···O intermolecular hydrogen bonds pack the molecules into infinite chains (Table 1, Fig.2).

### Experimental

The solution of succinic anhydride (0.01 mole) in toluene (25 ml) was treated dropwise with the solution of 3-chloro,4-methylaniline (0.01 mole) also in toluene (20 ml) with constant stirring. The resulting mixture was stirred for about one hour and set aside for an additional hour at room temperature for completion of the reaction. The mixture was then treated with dilute hydrochloric acid to remove the unreacted 3-chloro-4-methyl- aniline. The resultant title compound was filtered under suction and washed thoroughly with water to remove the unreacted succinic anhydride and succinic acid. It was recrystallized to constant melting point from ethanol. The purity of the compound was checked and characterized by its infrared and NMR spectra.

Needle like colorless single crystals used in X-ray diffraction studies were grown in ethanolic solution by slow evaporation of the solvent at room temperature.

### Refinement

The H atoms of the NH group and the OH group were located in a difference map and later restrained to N—H = 0.86 (2) Å and O—H = 0.82 (2) Å, respectively. The other H atoms were positioned with idealized geometry using a riding model with aromatic C—H = 0.93 Å and methylene C—H = 0.97 Å. All H atoms were refined with isotropic displacement parameters set at 1.2 *U*_eq_(C-aromatic, N) and 1.5 *U*_eq_(C-methyl).

### Crystal data

**Table:**

C_11_H_12_ClNO_3_	*Z* = 4
*M**_r_* = 241.67	*F*(000) = 504
Triclinic, *P*1	*D*_x_ = 1.414 Mg m^−^^3^
Hall symbol: -P 1	Mo *K*α radiation, λ = 0.71073 Å
*a* = 6.8788 (9) Å	Cell parameters from 1965 reflections
*b* = 7.9713 (9) Å	θ = 2.6–27.8°
*c* = 21.119 (3) Å	µ = 0.33 mm^−^^1^
α = 86.76 (1)°	*T* = 293 K
β = 86.48 (1)°	Needle, colourless
γ = 79.45 (1)°	0.42 × 0.14 × 0.08 mm
*V* = 1135.1 (3) Å^3^	

### Data collection

**Table:**

Oxford Diffraction Xcalibur diffractometer with a Sapphire CCD detector	4066 independent reflections
Radiation source: fine-focus sealed tube	2649 reflections with *I* > 2σ(*I*)
Graphite monochromator	*R*_int_ = 0.028
Rotation method data acquisition using ω and phi scans	θ_max_ = 25.3°, θ_min_ = 2.6°
Absorption correction: multi-scan (*CrysAlis RED*; Oxford Diffraction, 2009)	*h* = −8→7
*T*_min_ = 0.875, *T*_max_ = 0.974	*k* = −9→5
6890 measured reflections	*l* = −25→24

### Refinement

**Table:**

Refinement on *F*^2^	Primary atom site location: structure-invariant direct methods
Least-squares matrix: full	Secondary atom site location: difference Fourier map
*R*[*F*^2^ > 2σ(*F*^2^)] = 0.083	Hydrogen site location: inferred from neighbouring sites
*wR*(*F*^2^) = 0.159	H atoms treated by a mixture of independent and constrained refinement
*S* = 1.26	*w* = 1/[σ^2^(*F*_o_^2^) + (0.0164*P*)^2^ + 2.1147*P*] where *P* = (*F*_o_^2^ + 2*F*_c_^2^)/3
4066 reflections	(Δ/σ)_max_ < 0.001
303 parameters	Δρ_max_ = 0.23 e Å^−^^3^
4 restraints	Δρ_min_ = −0.29 e Å^−^^3^

### Special details

**Table:**

Experimental. CrysAlis RED (Oxford Diffraction, 2009) Empirical absorption correction using spherical harmonics, implemented in SCALE3 ABSPACK scaling algorithm.
Geometry. All e.s.d.'s (except the e.s.d. in the dihedral angle between two l.s. planes) are estimated using the full covariance matrix. The cell e.s.d.'s are taken into account individually in the estimation of e.s.d.'s in distances, angles and torsion angles; correlations between e.s.d.'s in cell parameters are only used when they are defined by crystal symmetry. An approximate (isotropic) treatment of cell e.s.d.'s is used for estimating e.s.d.'s involving l.s. planes.
Refinement. Refinement of *F*^2^ against ALL reflections. The weighted *R*-factor *wR* and goodness of fit *S* are based on *F*^2^, conventional *R*-factors *R* are based on *F*, with *F* set to zero for negative *F*^2^. The threshold expression of *F*^2^ > σ(*F*^2^) is used only for calculating *R*-factors(gt) *etc*. and is not relevant to the choice of reflections for refinement. *R*-factors based on *F*^2^ are statistically about twice as large as those based on *F*, and *R*- factors based on ALL data will be even larger.

### Fractional atomic coordinates and isotropic or equivalent isotropic displacement parameters (Å^2^)

**Table:**

	*x*	*y*	*z*	*U*_iso_*/*U*_eq_	
C1	0.9701 (7)	0.4350 (5)	0.2737 (2)	0.0373 (11)	
C2	0.8270 (7)	0.4875 (6)	0.3209 (2)	0.0395 (11)	
H2	0.6996	0.4647	0.3192	0.047*	
C3	0.8765 (7)	0.5746 (6)	0.3707 (2)	0.0406 (11)	
C4	1.0621 (8)	0.6124 (6)	0.3758 (2)	0.0462 (12)	
C5	1.2006 (7)	0.5579 (6)	0.3278 (2)	0.0508 (13)	
H5	1.3280	0.5803	0.3297	0.061*	
C6	1.1575 (7)	0.4713 (6)	0.2771 (2)	0.0492 (13)	
H6	1.2545	0.4376	0.2454	0.059*	
C7	0.7569 (7)	0.3702 (6)	0.1926 (2)	0.0442 (12)	
C8	0.7444 (8)	0.2452 (6)	0.1422 (2)	0.0520 (14)	
H8A	0.8542	0.2459	0.1111	0.062*	
H8B	0.7571	0.1310	0.1618	0.062*	
C9	0.5540 (8)	0.2863 (7)	0.1088 (3)	0.0643 (16)	
H9A	0.4443	0.2925	0.1402	0.077*	
H9B	0.5453	0.3979	0.0872	0.077*	
C10	0.5339 (9)	0.1589 (7)	0.0616 (3)	0.0625 (16)	
C11	1.1143 (8)	0.7015 (7)	0.4315 (2)	0.0641 (16)	
H11A	1.0990	0.6329	0.4698	0.077*	
H11B	1.0282	0.8103	0.4347	0.077*	
H11C	1.2491	0.7180	0.4258	0.077*	
N1	0.9276 (6)	0.3407 (5)	0.22252 (18)	0.0444 (10)	
H1N	1.019 (5)	0.261 (4)	0.210 (2)	0.053*	
O1	0.6196 (5)	0.4867 (4)	0.20492 (16)	0.0577 (10)	
O2	0.6511 (6)	0.0290 (5)	0.0539 (2)	0.0819 (14)	
O3	0.3768 (7)	0.2020 (6)	0.0292 (2)	0.1039 (18)	
H3O	0.370 (11)	0.124 (6)	0.006 (3)	0.125*	
Cl1	0.6908 (2)	0.63733 (18)	0.42917 (6)	0.0627 (4)	
C12	0.5122 (7)	1.0684 (5)	0.7315 (2)	0.0391 (11)	
C13	0.6607 (7)	0.9993 (6)	0.6890 (2)	0.0422 (12)	
H13	0.7919	1.0053	0.6953	0.051*	
C14	0.6131 (7)	0.9208 (5)	0.6365 (2)	0.0397 (11)	
C15	0.4223 (7)	0.9079 (6)	0.6246 (2)	0.0415 (11)	
C16	0.2766 (7)	0.9777 (7)	0.6688 (2)	0.0534 (14)	
H16	0.1457	0.9702	0.6628	0.064*	
C17	0.3185 (7)	1.0581 (6)	0.7213 (2)	0.0473 (12)	
H17	0.2168	1.1048	0.7497	0.057*	
C18	0.7041 (8)	1.1042 (7)	0.8223 (2)	0.0573 (15)	
C19	0.7225 (8)	1.2230 (7)	0.8742 (3)	0.0707 (18)	
H19A	0.6436	1.3344	0.8645	0.085*	
H19B	0.6696	1.1790	0.9141	0.085*	
C20	0.9289 (9)	1.2424 (8)	0.8816 (3)	0.0722 (18)	
H20A	0.9796	1.2907	0.8422	0.087*	
H20B	1.0086	1.1303	0.8893	0.087*	
C21	0.9521 (10)	1.3530 (7)	0.9343 (3)	0.0619 (16)	
C22	0.3714 (8)	0.8265 (7)	0.5666 (2)	0.0644 (16)	
H22A	0.4427	0.7112	0.5655	0.077*	
H22B	0.4072	0.8903	0.5292	0.077*	
H22C	0.2317	0.8261	0.5682	0.077*	
N2	0.5543 (6)	1.1566 (5)	0.78445 (19)	0.0485 (11)	
H2N	0.485 (6)	1.254 (4)	0.790 (2)	0.058*	
O4	0.8183 (7)	0.9688 (5)	0.8168 (2)	0.0951 (17)	
O5	0.8321 (7)	1.3876 (6)	0.9774 (2)	0.0946 (16)	
O6	1.1205 (9)	1.4050 (7)	0.9300 (2)	0.1004 (16)	
H6O	1.122 (11)	1.457 (8)	0.963 (2)	0.121*	
Cl2	0.8053 (2)	0.84014 (18)	0.58273 (6)	0.0599 (4)	

### Atomic displacement parameters (Å^2^)

**Table:**

	*U*^11^	*U*^22^	*U*^33^	*U*^12^	*U*^13^	*U*^23^
C1	0.038 (3)	0.036 (3)	0.037 (2)	0.001 (2)	−0.008 (2)	−0.010 (2)
C2	0.038 (3)	0.042 (3)	0.040 (3)	−0.007 (2)	−0.007 (2)	−0.009 (2)
C3	0.044 (3)	0.038 (3)	0.038 (3)	0.001 (2)	−0.005 (2)	−0.011 (2)
C4	0.056 (3)	0.040 (3)	0.045 (3)	−0.009 (2)	−0.013 (2)	−0.006 (2)
C5	0.040 (3)	0.056 (3)	0.059 (3)	−0.012 (2)	−0.009 (2)	−0.012 (3)
C6	0.046 (3)	0.056 (3)	0.044 (3)	0.000 (2)	−0.001 (2)	−0.015 (2)
C7	0.051 (3)	0.040 (3)	0.040 (3)	−0.001 (2)	−0.006 (2)	−0.014 (2)
C8	0.068 (4)	0.038 (3)	0.048 (3)	0.004 (2)	−0.020 (3)	−0.019 (2)
C9	0.057 (4)	0.069 (4)	0.068 (4)	0.001 (3)	−0.020 (3)	−0.037 (3)
C10	0.063 (4)	0.064 (4)	0.063 (4)	−0.002 (3)	−0.020 (3)	−0.032 (3)
C11	0.068 (4)	0.072 (4)	0.059 (3)	−0.017 (3)	−0.017 (3)	−0.025 (3)
N1	0.044 (3)	0.042 (2)	0.046 (2)	0.0057 (19)	−0.0109 (19)	−0.0200 (19)
O1	0.053 (2)	0.055 (2)	0.061 (2)	0.0148 (18)	−0.0208 (17)	−0.0312 (18)
O2	0.081 (3)	0.067 (3)	0.097 (3)	0.016 (2)	−0.042 (2)	−0.051 (2)
O3	0.088 (3)	0.095 (3)	0.126 (4)	0.029 (3)	−0.065 (3)	−0.073 (3)
Cl1	0.0659 (9)	0.0708 (9)	0.0511 (8)	−0.0068 (7)	0.0061 (6)	−0.0275 (7)
C12	0.047 (3)	0.030 (2)	0.041 (3)	−0.002 (2)	−0.009 (2)	−0.010 (2)
C13	0.030 (3)	0.049 (3)	0.048 (3)	−0.002 (2)	−0.006 (2)	−0.014 (2)
C14	0.041 (3)	0.034 (3)	0.043 (3)	−0.001 (2)	−0.003 (2)	−0.007 (2)
C15	0.041 (3)	0.044 (3)	0.042 (3)	−0.012 (2)	−0.004 (2)	−0.005 (2)
C16	0.034 (3)	0.072 (4)	0.057 (3)	−0.014 (3)	−0.008 (2)	−0.010 (3)
C17	0.038 (3)	0.055 (3)	0.048 (3)	−0.003 (2)	−0.001 (2)	−0.011 (2)
C18	0.059 (4)	0.055 (3)	0.057 (3)	0.006 (3)	−0.017 (3)	−0.028 (3)
C19	0.068 (4)	0.072 (4)	0.070 (4)	0.010 (3)	−0.024 (3)	−0.045 (3)
C20	0.082 (5)	0.066 (4)	0.071 (4)	−0.006 (3)	−0.019 (3)	−0.040 (3)
C21	0.073 (4)	0.061 (4)	0.055 (4)	−0.011 (3)	−0.021 (3)	−0.019 (3)
C22	0.066 (4)	0.082 (4)	0.054 (3)	−0.029 (3)	−0.010 (3)	−0.017 (3)
N2	0.049 (3)	0.041 (2)	0.053 (2)	0.0089 (19)	−0.011 (2)	−0.024 (2)
O4	0.105 (3)	0.072 (3)	0.097 (3)	0.048 (3)	−0.062 (3)	−0.058 (2)
O5	0.109 (4)	0.111 (4)	0.077 (3)	−0.045 (3)	0.005 (3)	−0.053 (3)
O6	0.121 (4)	0.126 (4)	0.071 (3)	−0.053 (3)	−0.001 (3)	−0.049 (3)
Cl2	0.0528 (8)	0.0685 (9)	0.0563 (8)	0.0000 (7)	0.0034 (6)	−0.0261 (7)

### Geometric parameters (Å, º)

**Table:**

C1—C6	1.378 (6)	C12—C13	1.375 (6)
C1—C2	1.384 (6)	C12—C17	1.380 (6)
C1—N1	1.424 (5)	C12—N2	1.426 (5)
C2—C3	1.385 (6)	C13—C14	1.387 (6)
C2—H2	0.9300	C13—H13	0.9300
C3—C4	1.376 (7)	C14—C15	1.375 (6)
C3—Cl1	1.746 (4)	C14—Cl2	1.743 (4)
C4—C5	1.380 (7)	C15—C16	1.387 (6)
C4—C11	1.502 (6)	C15—C22	1.507 (6)
C5—C6	1.382 (6)	C16—C17	1.383 (6)
C5—H5	0.9300	C16—H16	0.9300
C6—H6	0.9300	C17—H17	0.9300
C7—O1	1.223 (5)	C18—O4	1.220 (6)
C7—N1	1.344 (6)	C18—N2	1.334 (6)
C7—C8	1.516 (6)	C18—C19	1.515 (6)
C8—C9	1.499 (7)	C19—C20	1.475 (8)
C8—H8A	0.9700	C19—H19A	0.9700
C8—H8B	0.9700	C19—H19B	0.9700
C9—C10	1.492 (6)	C20—C21	1.491 (7)
C9—H9A	0.9700	C20—H20A	0.9700
C9—H9B	0.9700	C20—H20B	0.9700
C10—O2	1.202 (6)	C21—O5	1.200 (7)
C10—O3	1.297 (6)	C21—O6	1.297 (7)
C11—H11A	0.9600	C22—H22A	0.9600
C11—H11B	0.9600	C22—H22B	0.9600
C11—H11C	0.9600	C22—H22C	0.9600
N1—H1N	0.853 (19)	N2—H2N	0.843 (19)
O3—H3O	0.83 (2)	O6—H6O	0.83 (2)
			
C6—C1—C2	119.4 (4)	C13—C12—C17	119.5 (4)
C6—C1—N1	119.7 (4)	C13—C12—N2	121.0 (4)
C2—C1—N1	120.9 (4)	C17—C12—N2	119.5 (4)
C1—C2—C3	118.9 (4)	C12—C13—C14	119.4 (4)
C1—C2—H2	120.5	C12—C13—H13	120.3
C3—C2—H2	120.5	C14—C13—H13	120.3
C4—C3—C2	123.3 (4)	C15—C14—C13	123.0 (4)
C4—C3—Cl1	119.6 (3)	C15—C14—Cl2	119.1 (3)
C2—C3—Cl1	117.1 (4)	C13—C14—Cl2	117.8 (4)
C3—C4—C5	116.0 (4)	C14—C15—C16	116.0 (4)
C3—C4—C11	122.3 (5)	C14—C15—C22	122.7 (4)
C5—C4—C11	121.6 (5)	C16—C15—C22	121.3 (5)
C4—C5—C6	122.7 (5)	C17—C16—C15	122.5 (5)
C4—C5—H5	118.7	C17—C16—H16	118.7
C6—C5—H5	118.7	C15—C16—H16	118.7
C1—C6—C5	119.7 (4)	C12—C17—C16	119.6 (4)
C1—C6—H6	120.1	C12—C17—H17	120.2
C5—C6—H6	120.1	C16—C17—H17	120.2
O1—C7—N1	123.6 (4)	O4—C18—N2	123.1 (4)
O1—C7—C8	121.7 (4)	O4—C18—C19	121.3 (5)
N1—C7—C8	114.7 (4)	N2—C18—C19	115.6 (4)
C9—C8—C7	112.8 (4)	C20—C19—C18	112.7 (5)
C9—C8—H8A	109.0	C20—C19—H19A	109.1
C7—C8—H8A	109.0	C18—C19—H19A	109.1
C9—C8—H8B	109.0	C20—C19—H19B	109.1
C7—C8—H8B	109.0	C18—C19—H19B	109.1
H8A—C8—H8B	107.8	H19A—C19—H19B	107.8
C10—C9—C8	113.5 (4)	C19—C20—C21	113.7 (5)
C10—C9—H9A	108.9	C19—C20—H20A	108.8
C8—C9—H9A	108.9	C21—C20—H20A	108.8
C10—C9—H9B	108.9	C19—C20—H20B	108.8
C8—C9—H9B	108.9	C21—C20—H20B	108.8
H9A—C9—H9B	107.7	H20A—C20—H20B	107.7
O2—C10—O3	122.9 (5)	O5—C21—O6	122.6 (5)
O2—C10—C9	123.8 (5)	O5—C21—C20	124.6 (6)
O3—C10—C9	113.3 (5)	O6—C21—C20	112.8 (6)
C4—C11—H11A	109.5	C15—C22—H22A	109.5
C4—C11—H11B	109.5	C15—C22—H22B	109.5
H11A—C11—H11B	109.5	H22A—C22—H22B	109.5
C4—C11—H11C	109.5	C15—C22—H22C	109.5
H11A—C11—H11C	109.5	H22A—C22—H22C	109.5
H11B—C11—H11C	109.5	H22B—C22—H22C	109.5
C7—N1—C1	125.1 (4)	C18—N2—C12	125.3 (4)
C7—N1—H1N	118 (3)	C18—N2—H2N	118 (4)
C1—N1—H1N	117 (3)	C12—N2—H2N	116 (3)
C10—O3—H3O	109 (5)	C21—O6—H6O	105 (5)
			
C6—C1—C2—C3	−0.4 (7)	C17—C12—C13—C14	0.1 (7)
N1—C1—C2—C3	178.1 (4)	N2—C12—C13—C14	−177.3 (4)
C1—C2—C3—C4	0.1 (7)	C12—C13—C14—C15	0.0 (7)
C1—C2—C3—Cl1	−179.8 (3)	C12—C13—C14—Cl2	178.3 (4)
C2—C3—C4—C5	−0.1 (7)	C13—C14—C15—C16	−0.5 (7)
Cl1—C3—C4—C5	179.8 (4)	Cl2—C14—C15—C16	−178.8 (4)
C2—C3—C4—C11	−177.7 (5)	C13—C14—C15—C22	178.3 (5)
Cl1—C3—C4—C11	2.1 (7)	Cl2—C14—C15—C22	0.0 (7)
C3—C4—C5—C6	0.4 (8)	C14—C15—C16—C17	1.0 (8)
C11—C4—C5—C6	178.0 (5)	C22—C15—C16—C17	−177.8 (5)
C2—C1—C6—C5	0.6 (7)	C13—C12—C17—C16	0.3 (7)
N1—C1—C6—C5	−177.9 (4)	N2—C12—C17—C16	177.8 (5)
C4—C5—C6—C1	−0.6 (8)	C15—C16—C17—C12	−0.9 (8)
O1—C7—C8—C9	2.6 (8)	O4—C18—C19—C20	44.0 (9)
N1—C7—C8—C9	−178.3 (5)	N2—C18—C19—C20	−137.0 (6)
C7—C8—C9—C10	−176.6 (5)	C18—C19—C20—C21	−177.5 (5)
C8—C9—C10—O2	5.2 (9)	C19—C20—C21—O5	19.0 (9)
C8—C9—C10—O3	−174.7 (6)	C19—C20—C21—O6	−163.0 (6)
O1—C7—N1—C1	2.5 (8)	O4—C18—N2—C12	−3.1 (10)
C8—C7—N1—C1	−176.6 (4)	C19—C18—N2—C12	178.0 (5)
C6—C1—N1—C7	−141.8 (5)	C13—C12—N2—C18	−44.3 (8)
C2—C1—N1—C7	39.7 (7)	C17—C12—N2—C18	138.3 (6)

### Hydrogen-bond geometry (Å, º)

**Table:**

*D*—H···*A*	*D*—H	H···*A*	*D*···*A*	*D*—H···*A*
N1—H1*N*···O4^i^	0.85 (2)	2.05 (2)	2.882 (5)	165 (5)
O3—H3*O*···O2^ii^	0.83 (2)	1.83 (2)	2.654 (5)	173 (7)
N2—H2*N*···O1^iii^	0.84 (2)	2.07 (2)	2.891 (5)	165 (5)
O6—H6*O*···O5^iv^	0.83 (2)	1.90 (3)	2.703 (5)	164 (7)

Symmetry codes: (i) −*x*+2, −*y*+1, −*z*+1; (ii) −*x*+1, −*y*, −*z*; (iii) −*x*+1, −*y*+2, −*z*+1; (iv) −*x*+2, −*y*+3, −*z*+2.
